# N-terminal pro b-type natriuretic peptide (NT-pro-BNP) –based score can predict in-hospital mortality in patients with heart failure

**DOI:** 10.1038/srep29590

**Published:** 2016-07-14

**Authors:** Ya-Ting Huang, Yuan-Teng Tseng, Tung-Wei Chu, John Chen, Min-Yu Lai, Woung-Ru Tang, Chih-Chung Shiao

**Affiliations:** 1 Doctoral Candidate, Graduate Institute of Clinical Medical Science, College of Medicine, Chang Gung University. No. 259, Wenhua 1st Rd., Guishan Dist., Taoyuan City 33302, Taiwan, R.O.C.; 2 Department of Nursing, Department of Surgery, Saint Mary’s Hospital Luodong. No. 160, Zhongheng S. Rd., Luodong, Yilan 26546, Taiwan, R.O.C.; 3 Division of Cardiovascular Medicine, Department of Internal Medicine, Saint Mary’s Hospital Luodong. No. 160, Zhongheng S. Rd., Luodong, Yilan 26546, Taiwan, R.O.C.; 4 Division of Cardiovascular Surgery, Department of Surgery, Saint Mary’s Hospital Luodong. No. 160, Zhongheng S. Rd., Luodong, Yilan 26546, Taiwan, R.O.C.; 5 Graduate Institute of Nursing, Chang Gung University, Taiwan 259 Wen-Hwa 1st Road, Kwei-Shan, Tao-Yuan, Taiwan, ROC.; 6 Division of Nephrology in Department of Internal Medicine, Department of Surgery, Saint Mary’s Hospital Luodong. No. 160, Zhongheng S. Rd., Luodong, Yilan 26546, Taiwan, R.O.C.; 7 Saint Mary’s Medicine, Nursing and Management College. No. 100, Ln. 265, Sec. 2, Sanxing Rd., Sanxing Township, Yilan County 266, Taiwan, R.O.C.

## Abstract

Serum N-terminal pro b-type natriuretic peptide (NT-pro-BNP) testing is recommended in the patients with heart failure (HF). We hypothesized that NT-pro-BNP, in combination with other clinical factors in terms of a novel NT-pro BNP-based score, may provide even better predictive power for in-hospital mortality among patients with HF. A retrospective study enrolled adult patients with hospitalization-requiring HF who fulfilled the predefined criteria during the period from January 2011 to December 2013. We proposed a novel scoring system consisting of several independent predictors including NT-pro-BNP for predicting in-hospital mortality, and then compared the prognosis-predictive power of the novel NT-pro BNP-based score with other prognosis-predictive scores. A total of 269 patients were enrolled in the current study. Factors such as “serum NT-pro-BNP level above 8100 mg/dl,” “age above 79 years,” “without taking angiotensin converting enzyme inhibitors/angiotensin receptor blocker,” “without taking beta-blocker,” “without taking loop diuretics,” “with mechanical ventilator support,” “with non-invasive ventilator support,” “with vasopressors use,” and “experience of cardio-pulmonary resuscitation” were found as independent predictors. A novel NT-pro BNP-based score composed of these risk factors was proposed with excellent predictability for in-hospital mortality. The proposed novel NT-pro BNP-based score was extremely effective in predicting in-hospital mortality in HF patients.

Heart failure (HF) is a complex and fatal medical condition which progresses with increasing age and causes considerable morbidity and mortality, resulting in a tremendous burden on the healthcare system worldwide^1^,^2^. Around 20% of those over 65 years of age in the United States population has been hospitalized due to this entity. Meanwhile, cardiac disease stands as one of the top three leading causes of death in Taiwan in which cardiac disease and the more specific entity, HF, accounted for 11% and 2%, respectively, of all deaths in 2012^3^. Despite medical and technological advancement, the prognosis of HF remains poor with reported in-hospital mortality as high as 4–10%^2^,^4^,^5^,^6^,^7^,^8^,^9^,^10^. To develop treatment strategies and ameliorate patient outcomes, the identification of factors correlated with prognoses for HF patients is significant^4^,^5^,^6^,^7^,^8^,^9^,^10^.

In clinical practice, risk prediction models are useful in providing unique settings to predict prognoses in more particular patient groups. By using these models to identify patients at high risk for poor outcomes, the patients may receive benefits from the subsequent consistent monitoring and intervention from physicians^6^,^8^,^9^. In previous studies evaluating the prognoses of HF patients, several factors such as age, systolic blood pressure, serum blood urea nitrogen, creatinine, and sodium were demonstrated in influencing in-hospital mortality, and the proposed risk prediction models were thus of more precise predictability^4^,^5^,^6^,^7^,^8^,^9^. However, there are still potential limitations that need to be addressed. One is that the variables implemented in these models for risk prediction may not be available at the time of initial presentation to the hospital in real world practice^4^,^8^,^9^. Another limitation is that certain factors thought to influence patient outcomes were not put into the final prediction models. For example, b-type natriuretic peptide (BNP) is widely recognized as an outcome-predicting factor for HF patients, but it was not included in the risk prediction models because it was only available in less than 25% of the enrolled patients^5^,^7^.

Biomarkers such as natriuretic peptides have been suggested to be useful in determining the severity of disease and prognosis of clinical outcomes in patients with HF^11^. BNP and N-terminal pro-BNP (NT-pro-BNP) are peptides secreted by the cardiac ventricles in response to volume expansion and pressure load^11^. Increasing serum BNP and NT-pro-BNP levels grossly correlate with the severity of left ventricle dysfunction in both clinical and hemodynamic aspects^11^,^12^,^13^,^14^. Because of the different biological characteristics including half-life and *in-vitro* stability, as well as clearance mechanisms of these two peptides^15^, NT-pro-BNP is four-to-six folds higher than BNP in their steady-state levels although both peptides are released in equimolar amounts in circulation^13^,^15^,^16^,^17^. Nonetheless, the two peptides have similar diagnostic accuracies for differentially diagnosing patients with dyspnea^18^,^19^. Serum BNP and NT-pro-BNP tests were recommended as diagnostic tools for HF by the American Heart Association (AHA) in 2005 and 2009, respectively^20^. Therefore, both tests have now been widely used as screening and/or diagnostic tools for HF in patients presenting acute dyspnea^8^,^15^,^21^. Besides the diagnostic role, BNP and NT-pro-BNP are also reliable biomarkers for grading the severities and predicting the mortality risk in patients with HF^22^,^23^,^24^,^25^. The measurement of serum BNP is of great help in guiding decision-making with the therapeutic strategies which might further lead to different prognoses^8^,^11^.

Although BNP and NT-pro-BNP are of good prognosis-predictive ability for HF, other clinical factors may also play important roles in affecting outcomes. We hypothesized that NT-pro-BNP in combination with other relevant clinical factors may provide better predictive power for in-hospital mortality among patients with hospitalization-requiring HF. Furthermore, we conducted this study to experiment with proposing a novel NT-pro BNP-based scoring system for predicting in-hospital mortality.

## Result

A total of 1276 patients were screened during the study period. During the selection procedure, 990 patients were excluded due to a variety of factors, including: a lack of final diagnosis of HF at discharge, an age younger than 18, presence of severe chronic pulmonary diseases, the presence of decompensated hepatic diseases with ascites, and renal failure requiring renal replacement therapy. A total of 17 patients were additionally excluded due to a lack of echocardiography examinations. Finally, 269 patients (mean age, 74.5 ± 13.6 years; female, 53.9%) were included in this study. The mean and median levels of serum NT-pro BNP in all patients are 11530.9 ± 12062.0 pg/ml and 6494.0 pg/ml, respectively. Also, 139 patients (51.7%) had been admitted to the intensive care unit (ICU), and 48 patients (18.2%) received mechanical ventilator support. Overall, the mean length of total hospital stay was 12.1 ± 11.5 days, and 29 patients (10.8%) expired during the hospitalization (Table 1).

When setting α as 0.05 and odds ratio (OR) as 4.48, obtained from simple logistic regression evaluating the association between in-hospital mortality and NT-pro-BNP levels categorized by its best cut point by generalized additive models (GAM), the calculated power of logistic regression model in our study using the G-Power^26^ reached 1.00.

### NT-pro-BNP levels and in-hospital mortality

Comparing the patients without in-hospital mortality, those who expired during the hospitalization had significantly higher serum NT-pro-BNP levels (19829.0 ± 13542.9 pg/ml versus 10528.3 ± 11501.9 pg/ml in mean levels, and 15942.0 pg/ml versus 6013.0 pg/ml in median levels) (*z* value, −3.80; *p* < 0.001). The GAM revealed that the probability of in-hospital mortality initiated to elevate since NT-pro-BNP level of 0 pg/ml, and the best cut-off point of NT-pro-BNP level was 8100 pg/ml (Supplementary Fig. 1).

### NT-pro BNP-based score and in-hospital mortality

By using Pearson’s correlation analysis, 17 variables were significantly correlated with in-hospital mortality in our HF population, including age, New York Heart Association Functional Classification (NYHA Fc) of the heart, laboratory results upon initial hospitalization (serum sodium and serum NT-pro-BNP), ICU admission, mechanical ventilator and noninvasive positive pressure ventilator (NIPPV) support, experience of cardio-pulmonary resuscitation (CPR), vasopressor administration, comorbid diseases of hypertension and chronic kidney disease, infection during hospitalization, as well as oral medications during hospitalization (angiotensin converting enzyme inhibitors (ACEI), angiotensin receptor blocker (ARB), beta-blocker, Aldo. blocker, loop diuretics and digoxin) (Table 2).

After putting these 17 variables into a logistic regression model using the conditional forward stepwise procedure for multivariate analysis, 9 independent predictors of in-hospital mortality were exhibited. They included “serum NT-pro-BNP level > 8100 mg/dl” (adjusted OR = 6.65), “age > 79 years” (adjusted OR = 12.69), “without ACEI/ARB” (adjusted OR = 9.49), “without beta-blocker” (adjusted OR = 17.75), “without loop diuretics” (adjusted OR = 4.01), “with mechanical ventilator support” (adjusted OR = 9.47), “with NIPPV support” (adjusted OR = 6.18), “with vasopressor” (adjusted OR = 8.01), and “experience of CPR” (adjusted OR = 14.39) (Table 3 and Supplementary Table 1).

Subsequently, a novel NT-pro BNP-based score was proposed by using these predictors with individual weights, which were estimated by logistic regression analysis (Table 4). This formula contented the above-mentioned nine predictors with individual points of +1 to +3 and a total scoring range of 0–20 points. The average NT-pro BNP-based scores of all patients were 6.2 ± 3.8, which were significantly higher in patients with in-hospital mortality (12.2 ± 2.1) than those without (5.4 ± 3.2) (*p* < 0.0001) (data not shown in Tables).

**Figure 1:**
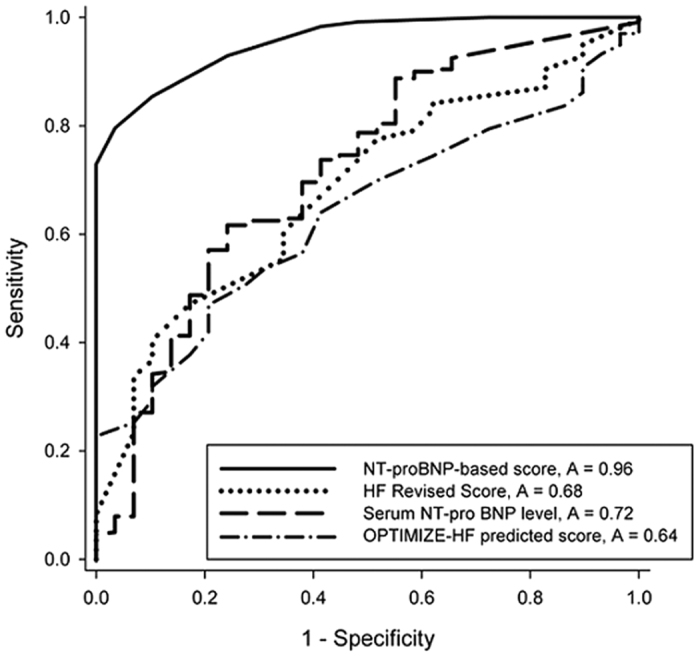
Better predictability for in-hospital mortality of NT-proBNP-based score comparing with serum NT-proBNP level, HF revised score, and OPTIMIZE-HF score. Note: Solid line denotes NT-pro BNP-based scores, with Area Under the Curve (AUC) of 0.96 (95% Confidence Interval (CI): 0.92–0.98, *p*-value < 0.0001); sensitivity of 0.97, specificity of 0.80, Positive Predictive Value (PPV) of 0.36, Negative Predictive Value (NPV) of 1.00, accuracy of 0.94. Dashed line denotes serum NT-pro-BNP levels, with AUC of 0.72 (95% CI: 0.66–0.77, p < 0.001), sensitivity of 0.76, specificity of 0.62, PPV of 0.19, NPV of 0.96, and accuracy of 0.64. Dotted line denotes HF revised score^9^, with AUC of 0.68 (95% CI: 0.59–0.78, *p* = 0.001). Dash-Dotted line denotes OPTIMIZE-HF score^4^, with AUC of 0.64 (95% CI: 0.55–0.73, *p* = 0.014). The AUC of NT-pro-BNP based score was higher than that of the serum NT-pro-BNP level, HF revised score^9^ and OPTIMIZE-HF score^4^ (*p* < 0.0001), while the AUC of serum NT-pro-BNP level was higher than the HF revised score^9^ and OPTIMIZE-HF score^4^ (*p* < 0.0001).

Finally, receiver operating characteristic (ROC) analysis for the predictability of in-hospital mortality was applied, which revealed a significantly better performance of the NT-pro BNP-based score (area under the curve (AUC), 0.96; 95% confidence interval (CI), 0.92–0.98; p < 0.0001; sensitivity, 0.97; specificity, 0.80; positive predictive value (PPV), 0.36; negative predictive value (NPV), 1.00; accuracy, 0.94) than that of serum NT-pro BNP level (AUC, 0.72; 95% CI, 0.66–0.77; *p* < 0.0001; sensitivity, 0.76; specificity, 0.62; PPV, 0.19; NPV, 0.96; accuracy, 0.64). Additionally, the AUC of NT-pro BNP-based scores was higher than that of the HF revised score^9^ (0.68; 95% CI, 0.59–0.77; *p* = 0.001) and Organized Program to Initiate Lifesaving Treatment in Hospitalized Patients With Heart Failure (OPTIMIZE-HF) score^4^ (0.64; 95% CI, 0.55–0.73; *p* = 0.014) (*p* < 0.001) (Fig. 1).

**Figure 2:**
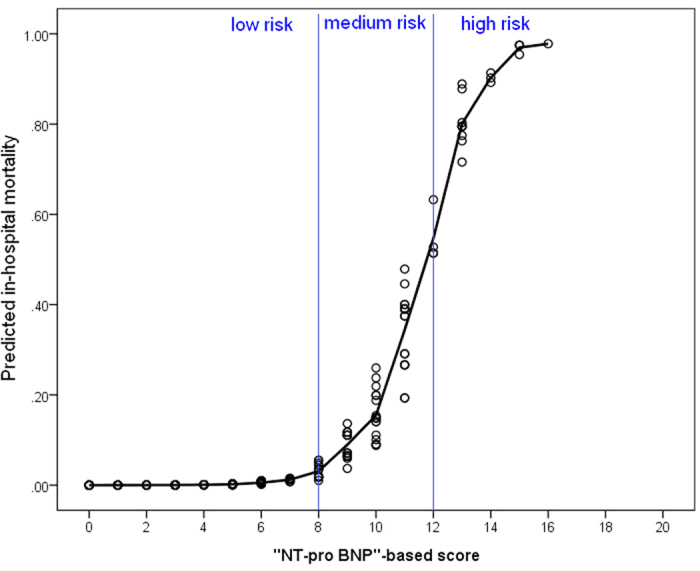
Association between the NT-pro BNP-based score and the probability of in-hospital mortality. Note: The NT-pro BNP-based score has a range of 0–20 points. According to the trend of increasing with the predicted in-hospital mortality, the NT-pro BNP-based score was categorized into low (<8 points), medium (≧8 to <12 points) and high risk (≧12 points), which represented the probability of in-hospital mortality of 0.52%, 21.70%, and 88.20%, respectively.

**Figure 3:**
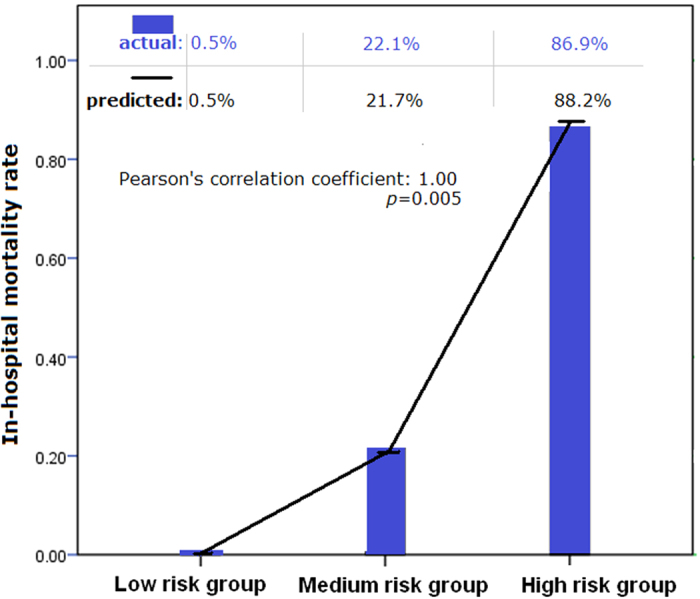
Comparison between the actual in-hospital mortality and the in-hospital mortality predicted by the NT-pro BNP-based scores in hospitalized HF patient. Note: The actual in-hospital mortality rates (bar) and the predicted morality rates (dotted line) had highly significant correlation (Pearson’s correlation coefficient = 1.00; *p* = 0.005).

Furthermore, by using the plot displaying the association between the “NT-pro-BNP”-based score and in-hospital mortality, we categorized the NT-pro BNP-based score into low (<8 points), medium (≧8 to <12 points) and high (≧12 points) risk groups. The predicted in-hospital mortality of the low, medium and high-risk groups were 0.5%, 21.7%, and 88.2%, respectively (Fig. 2). While applying the scores back to the current study population, the actual in-hospital mortality rates in low, medium and high-risk groups were 0.5%, 22.1%, and 86.9%. The actual in-hospital mortality and that predicted by the NT-pro BNP-based score was therefore significantly correlated. (Pearson’s correlation coefficient, 1.00; *p* = 0.005). (Fig. 3) The proposed model was demonstrated with an adequate calibration by using the Hosmer and Lemeshow test. (goodness-of-fit statistic of 2.88 with 8 degrees of freedom; *p* = 0.941) The appropriate fit of this proposed model was also shown in the plot of the change in deviance, which revealed only very few cases dispersing from the lines (Supplementary Figure 2).

### Comparison between HF with reduced ejection fraction (HFpEF) and HF with preserved ejection fraction (HFrEF)

Since HFrEF and HFpEF are considered two distinctly dissimilar entities in terms of therapy and survival^14^, we made an additional comparison in demographics, etiology, management and in-hospital mortality between the two patient groups.

In our studied population, 146 patients (54.3%) were HFrEF and 123 (45.7%) were HfpEF. Comparing with those with HFrEF, the population with HFpEF were older (76.7 ± 13.0 versus 72.5 ± 13.9 years, *p* = 0.01), consisted of more female patients (67.5% versus 42.5%, *p* < 0.001), presented higher serum NT-pro-BNP level at hospitalization (9072.3 ± 10113.8 versus 13602.3 ± 13168.1, *p* = 0.002), had longer length of hospital stay (13.9 ± 12.9 versus 10.7 ± 10.00, *p* = 0.026), showed lower proportions of presenting pulmonary edema (21.1% versus 37.0%, *p* = 0.005), and demonstrated lower proportions of taken oral diuretics (50.4% versus 69.9%, *p* = 0.001). Moreover, the NYHA Fc of the two groups was significant difference (*p* < 0.001). A majority of the patients with HFpEF was of NYHA Fc II (39.0%) and III (43.9%), while most of the patients with HFrEF were of NYHA Fc III (57.5%) and IV (28.8%). Nonetheless, other variables including vital signs and laboratory tests upon initial hospitalization, ICU admission, mechanical ventilator support, NIPPV support, vasopressors, CPR experience, drug history with ACEI/ARB, beta-blocker, aldosterone receptor antagonists, statins, as well as in-hospital mortality, were not statistically different between the patients with HFrEF and HFpEF. As for the proposed NT-pro-BNP score, it revealed accurate and equal predictive ability for in-hospital mortality in both groups (both AUCs of ROC were 0.96; Supplementary Figure 3).

## Discussion

Previously, several scores^4^,^5^,^6^,^7^,^8^,^9^ for predicting prognoses of HF patients had been proposed but none of them took serum NT-pro-BNP level as a risk factor. The current study is the first one to propose a novel NT-pro BNP-based score for in-hospital mortality in hospitalization-requiring HF patients. In our single-centered HF cohort, the score exhibited an excellent predictive power which was even better than HF revised score^9^ and OPTIMIZE-HF score^4^. This scoring formula was composed of 9 clinical predictors, which were weighted by logistic regression method. These predictors included “serum NT-pro-BNP level > 8100 mg/dl,” “age > 79 years,” “without ACEI/ARB,” “without beta-blocker,” “without loop diuretics,” “with mechanical ventilator support,” “with NIPPV support,” “with vasopressor,” and “experience of CPR.” The predictors in current NT-pro BNP-based scores were distinct from those in previous prognosis-predicting models in hospitalized HF patients^5^,^9^,^4^,^8^. There are several explanations for the differences of the current NT-pro BNP-based score from others.

The first important explanation is the thorough application of NT-pro-BNP test in the current study. In most of the scoring system, such as Acute Decompensated Heart Failure National Registry (ADHERE) risk score^5^, OPTIMIZE-HF risk score^4^, Get with the guidelines program (GWTG)-HF risk score^8^, Acute Heart Failure Database (AHEAD) registry^7^ and HF revised score^9^, the NT-pro-BNP level proved more difficult in being taken into consideration because its data was available in only about half of the participants in these studies.

Since the serum NT-pro BNP test has been recommended as a diagnostic tool for HF^20^, the population of HF patients diagnosed with the serum NT-pro BNP criteria in the current study were probably different from the HF populations diagnosed without the serum NT-pro BNP criteria in these studies^5^,^4^,^8^,^7^,^9^. Besides, NT-pro-BNP has also been proven as a reliable prognosis-predictive factor in HF patients^22^,^23^,^24^,^25^,^27^. Thus, any risk predictive scoring system that does not evaluate this major risk factor may result in an altered final predictive model and subsequent inaccuracy for outcome predicting.

Previously, several variables such as heart rate >140 bpm, creatinine clearance rate <60 ml/min/1.73m2, and serum sodium <130 mEq/L were exhibited as predictors for mortality. However in the current study, serum sodium <130 mEq/L (adjusted OR = 3.74; *p* = 0.005), heart rate >140 bpm (adjusted OR = 6.25; *p* = 0.008), and creatinine clearance rate <60 ml/min/1.73m2 (adjusted OR = 2.90; *p* = 0.045) were indeed disclosed as independent predictors before other variables, including serum NT-pro BPN level, that were inserted into the multivariate analysis. Conversely, the predictive abilities of the three variables were overtaken and replaced after placing a number of more powerful predictors, including the NT-pro-BNP level, into the multivariate comparison in the later steps (Supplementary Table 2).

In addition, previous studies tended to assess basic characteristics or clinical variables at initial hospitalization - rather than the procedure or pharmacologic intervention during the hospitalization - as potential risk factors^4^,^5^,^8^,^9^. In fact, the procedure itself or intervention such as medications, non-invasive or invasive ventilation support, and administration of vasopressor or experience of CPR also play important roles influencing in-hospital mortality among hospitalized HF patients^7^,^10^,^28^. According to the American College of Cardiology (ACC)/AHA guideline of HF pharmacological treatment, ACEI/ARB, and beta-blocker are suggested for decreasing mortality and improving symptoms in HF patients^20^. The OPTIMIZE-HF study^4^ demonstrated a lower risk of in-hospital mortality in patients taking ACEIs or beta-blockers at the time of admission, and similar results in acute HF patients were also found by the clinical quality improvement network (CQIN) investigators^29^. Aside from ACEI, ARBs are recommended in patients with systolic dysfunctional HF accompanied by current or prior symptoms who are ACEI intolerant by the ACC/AHA, to reduce morbidity and mortality^20^. Consistent with previous studies, the current study demonstrated the protective role of ACEI/ARB or beta-blocker impacting in-hospital mortality. The patients who took beta-blockers or ACEI/ARB during hospitalization had a lower risk of in-hospital mortality.

Theoretically, diuretics can increase urinary sodium excretion and decrease physical signs of fluid retention in patients with HF, resulting in improved symptoms and exercise tolerance. But the direct effects of diuretics on morbidity and mortality were not known previously^20^,^30^. Nevertheless, the current study also showed the protective effect of diuretics regarding in-hospital mortality in the HF population.

Non-invasive or invasive ventilation support, administration of vasopressor, and experience of CPR were considered as prognostic endpoints or dependent variables in several studies^10^,^31^. Spinar *et al*.^7^ discovered that mechanical ventilation support (OR = 15.30; p < 0.05) and use of vasopressors (OR = 5.90; p < 0.05) were associated with higher in-hospital mortality. Sadhu *et al*.^28^ disclosed that in-hospital mortality rate increased to as high as 75% after CPR for in-hospital cardiac arrest among hospitalized patients with HF, with a majority of patients dying within the first post-CPR day. Taking the results of the current study and previous investigations together, the use of non-invasive or invasive ventilation, administration of vasopressor and CPR should be considered as adverse prognostic factors and included into the risk predictive scores of increasing predictability.

It is worth mentioning that left ventricular ejection flow (LVEF) did not exhibit its predictive ability for in-hospital mortality in our study, whether in the entire HF population or subgroup with HFrEF or HFpEF. Actually, while LVEF was considered as a predictor of mortality in many studies, there were still other studies that showed dissimilar results^8^,^9^. The population in current study consisted of hospitalized HF patients who might have an acute component of HF rather than chronic stable HF. It is probable that the LVEF determined by echocardiography during the hospitalization could reflect an acutely deteriorated heart function rather than a chronic stable status; thus, it could not predict prognosis. Similarly, the HFrEF or HFpEF defined by the LVEF and evaluated during hospitalization may also not truly reflect the chronic stable heart function of the patients. This may further explain why the pharmacologic intervention, such as the use of ACEI/ARB or beta-blocker and even in-hospital mortality, was not statistically different between the two groups with HFrEF and HFpEF.

### Limitations

Several limitations of the current study should be addressed. First, the retrospective nature of the study was subjected to bias. Second, the current study enrolled HF patients who probably had acute components and fulfilled the age-specific serum NT-pro BNP cut-points. Those (such as obese patients) with clinical HF but lower than expected serum NT-pro-BNP levels would be excluded from this study. The results from the current study may not be applied to all HF patients, and the predictability of NT-pro-BNP was probably inflating. Third, the investigation was mainly limited to exploring the relationship between clinical prognosis and one serum NT-pro-BNP level at initial admission. The serial changes of serum NT-pro-BNP levels after management during hospitalization were not taken into consideration. Fourth, this is a single-center study without an external validation, which is its biggest limitation. Although we have done some cross-validation using the Hosmer and Lemeshow test to demonstrate the goodness-of-fit statistic of the HF revised score and OPTIMIZE-HF risk score in our study cohort, the finding that the proposed NT-pro BNP-based score has better predictability for in-hospital mortality than the serum NT-pro-BNP level, HF revised score and OPTIMIZE-HF score in our study cohort could not become a claim applying to other HF cohorts. Further multicentered, prospective research is warranted to confirm the predictive value of the proposed NT-pro BNP-based scores in all HF patients.

## Conclusion

In the current single-centered cohort, serum NT-pro BNP testing is not only a good diagnostic tool but also an adequate prognostic marker for hospitalized HF patients. The current study proposed a novel NT-pro BNP-based score with excellent predictability for in-hospital mortality in HF patients.

## Method

### Study population

This retrospective study was conducted during the period from January 1, 2011, to December 31, 2013, in a regional teaching hospital in the eastern part of Taiwan.

All adult hospitalized patients were eligible for this study once they met the following criteria: (1) had serum NT-pro BNP drawn within 24 hours upon hospitalization, which had reached the predefined age-specific cut-points; (2) had undergone echocardiography during the hospitalization; and (3) had final diagnosis of HF with International Classification of Diseases (ICD)-9 code of 428, 428.0, 428.1, and 428.9 at their discharge. The age-specific serum NT-pro BNP cut-points which had been proven to identify acute HF patients were as follows: (1) >1800 pg/ml in patients over 75 years; (2) >900 pg/ml in patients aged 50–75 years; and (3) >450 pg/ml in patients below 50 years^32^,^33^,^34^,^35^,^36^,^37^,^38^. The exclusion criteria included patients less than 18 years of age, along with those who had severe chronic pulmonary diseases (i.e. forced expiratory volume in one second < 1 liter in pulmonary function), decompensated hepatic diseases with ascites, and/or renal failure requiring renal replacement therapy. For those with more than one hospitalization, which was eligible with the inclusion and exclusion criteria during the study period, only the first hospitalization was included into the current study.

Information gathered from patients’ medical charts included baseline demographic data, comorbid diseases, Charlson combine scores, etiologies of HF, and NYHA Fc of the heart, along with other clinical variables including vital signs and laboratory results upon initial hospitalization, oral medications during hospitalization, LVEF from echocardiography examinations, chest roentgenogram, and atrial fibrillation (Af) from electrocardiogram, length of hospital stays, and in-hospital mortality. HFrEF and HFpEF were defined as LVEF <45% and ≧45%, respectively^14^.

In those admitted to ICU, additional information such as the implementation of a mechanical ventilator or NIPPV support, the experience of CPR, as well as the length of a mechanical ventilator or NIPPV support, ICU stays, and vasopressors support was also documented.

### Ethical approval

The Institutional Review Board of Saint Mary’s Hospital Luodong reviewed and approved this study (No. SMHIRB-103004). The study was carried out in accordance with the approved guidelines. Informed consents were waived since there was neither breach of privacy nor possible interference with clinical decisions.

### Statistical analysis

The statistical analyses and plot drawing were performed using the Scientific Package for Social Science (PASW Statistics for Windows, Version 22.0, Chicago: SPSS Inc), R 2.3.4 (R Foundation for Statistical Computing, Vienna, Austria) software and Sigma Plot software Version 12.5. Data are reported as mean ± standard deviation (SD) for continuous variables or case number (percentage) of non-missing values for categorical variables. The Mann-Whitney test was used to compare continuous variables between survivors and non-survivors. While the independent t-test and Chi-square test were used to compare continuous and categorical variables between HFrEF and HFpEF^14^.

The main statistical analyses were listed below: (1) All variables were put into Pearson’s correlation test to evaluate the correlation between in-hospital mortality and these variables. (2) The variables which exhibited significant correlation with in-hospital mortality in Pearson’s correlation test were put into the logistic regression model, using the conditional forward stepwise procedure for multivariate analysis to investigate their regression coefficient, OR, and *p*-value. The elimination criterion for the multivariate analysis was set at p > 0.05. The continuous variables would be transformed into categorical variables in advance by using their best cut points for measuring the probability of death by GAM. In addition, a bootstrap simulation (*2000), which was proposed for estimating sampling distributions and associated statistics for regression variables in multivariate models^39^, was used to verify the internal validity of this analysis. Resampling procedures were performed and applied to an appropriate joint distribution to estimate covariance matrices, make bias corrections, and construct CIs. (3) An NT-pro BNP-based scoring formula was created using the regression coefficient identified in the multivariate modeling. The scores of the individual predictors were composed of the arithmetic sum of b coefficients derived from logistic regression analysis, including all independent predictors after each numerical rounding. The calibration of the model was evaluated by the Hosmer and Lemeshow Goodness-of-Fit test and the plot of the change in deviance^40^. (4) ROC curve with AUC, as well as sensitivity, specificity, PPV, NPV, and accuracy were used to exam the predictive ability for in-hospital mortality of both the serum NT-pro-BNP level and the NT-pro BNP-based score.

Additionally, the two well-known prognostic scores, namely, HF revised score^9^ and OPTIMIZE-HF risk score^4^, which were proven with good fits (goodness-of-fit statistic with 8 degrees of freedom of 9.436 (p = 0.307) for HF revised score and of 10.84 (p = 0.211) for OPTIMIZE-HF risk score) in our study cohort by the Hosmer and Lemeshow test, were applied to the current study population for predictive power comparisons using the method of Hanley and McNeil^41^. In addition, the ROC curve with AUC has used to exam the predictive ability for in-hospital mortality of both HFrEF and HFpEF groups. Finally, plots were drawn to compare the association between the risk of in-hospital mortality and the NT-pro BNP-based scores. In all statistical analyses, a two-sided *p* ≦ 0.05 was considered statistically significant.

## Additional Information

**How to cite this article**: Huang, Y.-T. *et al*. N-terminal pro b-type natriuretic peptide (NT-pro-BNP) –based score can predict in-hospital mortality in patients with heart failure. *Sci. Rep.* **6**, 29590; doi: 10.1038/srep29590 (2016).

## Supplementary Material

Supplementary Information

## Figures and Tables

**Table 1:** Basic characteristics and clinical variables of participants (n = 269).

Variables	Total participants (n = 269)
Gender, female	145 (53.9)
Age	74.5 ± 13.6 (26–101)
BMI	23.6 ± 5.1 (26–101)
Cigarette smoking	203 (75.5)
HTN	93 (34.6)
NYHA Fc	
II	68 (25.3)
III	138 (51.3)
IV	63 (23.4)
Cause of HF	
VHD	51 (19.0)
DCM	23 (8.6)
IHD	135 (50.2)
RHD	6 (2.2)
HCVD	16 (5.9)
Infection during hospitalization	138 (51.3)
Comorbid diseases	
CLD	57 (21.2)
DM	115 (42.8)
CVA	34 (12.6)
CKD	104 (38.7)
Charlson combine scores	7.1 ± 2.7 (1–14)
Medication during hospitalization	
ACEI/ARB	146 (53.6)
Beta-blocker	96 (35.7)
Ald.–blocker	74 (27.5)
Loop-diuretic	164 (61.0)
Digoxin	40 (14.9)
Vital sign at initiation of hospitalization	
HR	96.3 ± 23.7 (42–162)
RR	22.9 ± 5.4 (10–51)
SBP	138.9 ± 31.6 (59–231)
DBP	82.2 ± 18.3 (38–149)
Blood test at initiation of hospitalization	
Serum NT-pro BNP level	11530.9 ± 12062.0 (507.1–83097.0)
WBC	10.1 ± 4.9 (2.9–39.9)
Hb	11.5 ± 2.5 (5.3–20.1)
Na	137.7 ± 5.9 (118–157)
K	4.1 ± 0.9 (2.3–7.4)
CCR	54.4 ± 34.3 (5.2–221.6)
EKG-Af	87 (32.3)
CXR-pulmonary edema	75 (27.9)
LVEF	48.9 ± 17.0 (9–87)
HFrEF	146 (54.3%)
HFpEF	123 (45.7%)
In-hospital mortality	29 (10.8)
Length of hospital stay	12.1 ± 11.5 (1–83)
ICU admission	139 (51.7)
Length of ICU stay	5.7 ± 5.8 (1–28)
Mechanical ventilator	49 (18.2)
Length of ventilator support	10.8 ± 9.2 (1–57)
Use of NIPPV	28 (10.4)
Length of NIPPV	4.54 ± 4.5 (1–18)
Use of vasopressor	33 (12.3)
CPR	13 (4.8)

Note: data was expressed as mean ± standard deviation (range) or n (%).

Abbreviations: ACEI = Angiotensin converting enzyme inhibitors, Af = Atrial fibrillation, Ald. –blocker = aldosterone receptor blocker, ARB = Angiotensin receptor blocker, Ald. blocker = Aldosterone receptor blocker, BMI = Body Mass Index, CCR = Creatinine clearance, CKD = Chronic kidney disease, CLD = chronic lung disease, CPR = Cardiopulmonary Resuscitation, CVA = cerebral vascular accident, CXR = chest radiograph, DBP = Diastolic blood pressure, DCM = Dilated cardiomyopathy, DM = Diabetes mellitus, EKG = Electrocardiogram, HF = Heart failure, Hb = Hemoglobin, HTN = Hypertension, HCVD = hypertensive cardiovascular disease, HFrEF = Heart failure with reduced ejection fraction, HFpEF = Heart failure with preserved ejection fraction, HR = Heart rate, IHD = ischemic heart disease, ICU = Intensive care unit, K = Potassium, LVEF = Left ventricular ejection fraction, Na = Sodium, NIPPV = Noninvasive positive pressure ventilator, NYHA Fc = New York Heart Association Functional Classification, RHD = Rheumatic heart disease, RR = Respiratory rate, SBP = Systolic blood pressure, WBC = white blood cell count, VHD = Valvular heart disease.

**Table 2:** The 17 variables with significant correlation with in-hospital mortality risk.

	In-hosp mortality	Age	NYHA	Na	NT-pro BNP	ICU	Vaso-pressors	CPR	HTN	CKD	Infection	Ventilator	NIPPV	ACE/ARB	Beta blocker	Aldo. blocker	Loop diuretic	Digox
In-hosp mortality	1.00																	
Age	0.12^*^	1.00																
NYHA	0.22^***^	0.058	1.00															
Na	−0.15^*^	−0.10	−0.08	1.00														
NT-pro BNP	0.23^***^	0.23^***^	0.39^***^	−0.12	1.00													
ICU	0.22^***^	0.05	0.22^***^	−0.13^*^	0.14^*^	1.00												
Vasopressors	0.38^***^	−0.06	0.14^*^	−0.01	0.04	0.32^***^	1.00											
CPR	0.37^***^	−0.18^**^	0.18^**^	0.06	0.06	0.18^**^	0.39^***^	1.00										
HTN	−0.15^*^	−0.04	−0.07	0.09	−0.02	0.03	−0.13^*^	−0.02	1.00									
CKD	0.14^*^	0.11	0.05	−0.17^**^	0.27^***^	0.11	0.03	−0.04	0.05	1.00								
Infection	0.15^*^	0.25^***^	0.15^*^	−0.17^**^	0.10	0.34^***^	0.14^*^	0.05	−0.18^**^	0.15^*^	1.00							
Ventilator	0.33^**^	−0.05	0.30^**^	−0.07	0.13^*^	0.44^**^	0.41^**^	0.43^**^	−0.08	−0.04	0.21^**^	1.00						
NIPPV	0.16^*^	0.13^*^	0.13^*^	−0.05	0.10	0.26^**^	0.02	−0.02	0.03	0.13^*^	0.21^**^	−0.16^**^	1.00					
ACE/ARB	−0.28^***^	−0.03	−0.02	0.24^***^	−0.12	−0.20^**^	−0.11	−0.07	0.17^**^	−0.22^***^	−0.15^*^	−0.09	−0.15	1.00				
Beta blocker	−0.23^***^	−0.18^**^	0.04	−0.03	−0.05	−0.15^*^	−0.18^**^	−0.10	0.13^*^	−0.00	−0.13^*^	−0.05	−0.15^*^	0.26^***^	1.00			
Aldo. blocker	−0.16^**^	−0.14^*^	0.08	0.09	−0.00	−0.15^*^	−0.08	−0.06	0.01	−0.13^*^	−0.12	−0.08	−0.10	0.18^**^	0.10	1.00		
Loop diuretic	−0.26^***^	−0.01	0.02	0.13^*^	0.12^*^	−0.21^**^	−0.21^***^	−0.10	0.18^**^	0.041	−0.17^**^	−0.22^**^	−0.10	0.15^*^	0.14^*^	0.14^*^	1.00	
Digox	−0.15^*^	−0.00	0.06	0.13^*^	−0.05	−0.08	−0.03	−0.09	−0.06	−0.053	0.01	−0.09	−0.01	0.20^**^	0.02	0.16^**^	0.10	1.00

Note: The correlation coefficient was tested by Pearson’s correlation test.

Abbreviations: ACEI = Angiotensin converting enzyme inhibitors, ARB = Angiotensin receptor blocker, Ald. blocker = Aldosterone receptor blocker, NIPPV = Noninvasive positive pressure ventilator, NT-pro BNP = Serum N-terminal-pro-B-type natriuretic peptide, CKD = Chronic kidney disease, Digox = Digoxin, HTN = Hypertension, Na  = blood sodium level, ICU = Intensive Care Unit, CPR = Cardiopulmonary Resuscitation, NYHA Fc = New York Heart Association Functional Classification. *p ≦ 0.05; **p ≦ 0.01; ***p < 0.001.

**Table 3:** The 9 independent predictors of in-hospital mortality.

Predictors	Adjusted OR	95% CI	*p*-value
**NT-pro BNP > 8100 pg/ml**			
No	Reference		
Yes	6.65	1.71–25.95	0.002
**Age > 79 years**			
No	Reference		
Yes	12.69	2.60–61.86	0.001
**Loop diuretics**			
Yes	Reference		
No	4.01	1.14–14.08	0.025
**Beta-blocker**			
Yes	Reference		
No	17.75	1.59–198.65	0.003
**ACEI/ARB**			
Yes	Reference		
No	9.49	2.13–42.21	0.002
**Vasopressor**			
No	Reference		
Yes	8.01	1.77–36.17	<0.001
**Mechanical ventilator**			
No	Reference		
Yes	9.47	2.06–43.54	0.001
**NIPPV**			
No	Reference		
Yes	6.18	1.35–28.21	0.028
**Experience of CPR**			
No	Reference		
Yes	14.39	1.63–127–28	0.003

Note: The 17 predictors which exhibited significant correlation with in-hospital mortality in Pearson’s correlation test (shown in Table 2) were put into the logistic regression model using the conditional forward stepwise procedure for multivariate analysis, with an elimination criterion of *p* > 0.05, to investigate their regression coefficient, OR, and *p*-value. The continuous variables would be transformed into categorical variables by using their best cut points for measuring the probability of death by generalized additive models. Bootstrap approach for logistic regression; 2000 resampling. The Hosmer and Lemeshow test demonstrated an adequate calibration of the proposed model (goodness-of-fit statistic 2.88 with 8 degrees of freedom (*p* = 0.941).

Abbreviations: ACEI = Angiotensin converting enzyme inhibitors, ARB = Angiotensin receptor blocker, CI = Confidence interval, CPR = Cardiopulmonary Resuscitation, NIPPV = Noninvasive Positive Pressure Ventilators; OR = odd’s ratio.

**Table 4:** Formula of the NT-pro BNP-based scoring system.

Risk factors	Points
NT-pro BNP level > 8100 mg/dl	+2
Age > 79 years	+3
Without ACEI/ARB	+2
Without beta-blocker	+3
Without loop diuretics	+1
With MV support	+2
With NIPPV support	+2
With vasopressor	+2
Experience of CPR	+3
Total scores (0–20 points)	

Note: The formula was created using the regression coefficient identified in the multivariate modeling. The scores of the individual predictors were composed of the arithmetic sum of b coefficients derived from logistic regression analysis including all independent predictors after each numerical rounding.

Abbreviations: ACEI = Angiotensin converting enzyme inhibitors, ARB = Angiotensin receptor blocker, CPR = Cardiopulmonary Resuscitation, MV = mechanical ventilator, NIPPV = Noninvasive Positive Pressure Ventilators, NT-pro BNP = N-terminal-pro-B-type natriuretic peptide.
