# Corrigendum: N-terminal pro b-type natriuretic peptide (NT-pro-BNP) –based score can predict in-hospital mortality in patients with heart failure

**DOI:** 10.1038/srep32902

**Published:** 2016-09-14

**Authors:** Ya-Ting Huang, Yuan-Teng Tseng, Tung-Wei Chu, John Chen, Min-Yu Lai, Woung-Ru Tang, Chih-Chung Shiao

Scientific Reports 6: Article number: 2959010.1038/srep29590; published online: 07 14 2016; updated: 09 14 2016

In the original version of this Article, there were errors in Affiliation 2 and 6 which were incorrectly listed as ‘Department of Nursing, Department of Surgery, Saint Mary’s Hospital Luodong. No. 160, Zhongheng S. Rd., Luodong, Yilan 26546, Taiwan, R.O.C.’ and ‘Division of Nephrology in Department of Internal Medicine, Department of Surgery, Saint Mary’s Hospital Luodong. No. 160, Zhongheng S. Rd., Luodong, Yilan 26546, Taiwan, R.O.C.’ respectively. The correct affiliations are listed below.

Affiliation 2

Department of Nursing, Saint Mary’s Hospital Luodong. No. 160, Zhongheng S. Rd., Luodong, Yilan 26546, Taiwan, ROC.

Affiliation 6

Division of Nephrology, Department of Internal Medicine, Saint Mary’s Hospital Luodong. No. 160, Zhongheng S. Rd., Luodong, Yilan 26546, Taiwan, ROC.

These errors have now been corrected in the PDF and HTML versions of the Article.

