# Corrigendum: N-terminal pro b-type natriuretic peptide (NT-pro-BNP) –based score can predict in-hospital mortality in patients with heart failure

**DOI:** 10.1038/srep46866

**Published:** 2017-06-30

**Authors:** Ya-Ting Huang, Yuan-Teng Tseng, Tung-Wei Chu, John Chen, Min-Yu Lai, Woung-Ru Tang, Chih-Chung Shiao

Scientific Reports 6: Article number: 2959010.1038/srep29590; published online: 07 14 2016; updated: 06 30 2017

In this Article, Affiliation 7 was incorrectly listed as ‘Saint Mary’s Medicine, Nursing and Management College. No. 100, Ln. 265, Sec. 2, Sanxing Rd., Sanxing Township, Yilan County 266, Taiwan, R.O.C’. The correct affiliation is listed below:

Saint Mary’s Junior College of Medicine, Nursing and Management. No. 100, Ln. 265, Sec. 2, Sanxing Rd., Sanxing Township, Yilan County 266, Taiwan, R.O.C.

This error has now been corrected in the PDF and HTML versions of this Article.

