# Supplementary material for: N-terminal pro b-type natriuretic peptide (NT-pro-BNP) –based score can predict in-hospital mortality in patients with heart failure
Source: Sci Rep. 2016 Jul 14;6:29590. doi: 10.1038/srep29590 (PMC4944149; doi:10.1038/srep29590)

**N-terminal pro b-type natriuretic peptide (NT-pro-BNP) –based score can predict in-hospital mortality in patients with heart failure**

Ya-Ting Huang, RN, MN, Yuan-Teng Tseng, MD, Tung-Wei Chu, MD, John Chen, MD, Min-Yu Lai, RN, Woung-Ru Tang, Ph.D., and Chih-Chung Shiao, MD.

**Supplementary information**

**Supplementary table 1. Steps of determining independent predictors for in-hospital mortality, model 1.**

|  |  | **Adjusted OR** | **95% CI** | **p-value** |
| --- | --- | --- | --- | --- |
| **1** | With vasopressor | 10.86 | 4.57-25.81 | <0.001 |
|  | Constant | 0.07 |  | <0.001 |
| **2** | Without ACEI/ARB | 8.93 | 2.86-27.93 | <0.001 |
|  | With vasopressor | 10.69 | 4.14-27.61 | <0.001 |
|  | Constant | 0.02 |  | <0.001 |
| **3** | NT-pro BNP>8100 pg/ml | 5.82 | 2.01-16.88 | <0.001 |
|  | Without ACEI/ARB | 9.45 | 2.85-31.35 | <0.001 |
|  | With vasopressor | 14.17 | 4.91-40.89 | <0.001 |
|  | Constant | 0.01 |  | <0.001 |
| **4** | NT-pro BNP>8100 pg/ml | 7.06 | 2.21-22.50 | 0.001 |
|  | With MV support | 4.71 | 1.64-13.49 | 0.004 |
|  | Without ACEI/ARB | 9.32 | 2.78-31.27 | <0.001 |
|  | With vasopressor | 7.80 | 2.47-24.62 | <0.001 |
|  | Constant | 0.00 |  | <0.001 |
| **5** | Age >79 years | 5.15 | 1.62-16.43 | 0.006 |
|  | NT-pro BNP>8100 pg/ml | 6.53 | 1.97-21.65 | 0.002 |
|  | With MV support | 6.11 | 2.01-18.57 | 0.001 |
|  | Without ACEI/ARB | 11.74 | 3.31-41.66 | <0.001 |
|  | With vasopressor | 11.90 | 3.46-40.98 | <0.001 |
|  | Constant | 0.00 |  | <0.001 |
| **6** | Age >79 years | 5.89 | 1.78-19.49 | 0.004 |
|  | NT-pro BNP>8100 pg/ml | 6.53 | 1.86-22.89 | 0.003 |
|  | With MV support | 11.81 | 3.23-43.25 | <0.001 |
|  | With NIPPV support | 6.84 | 1.72-27.24 | 0.006 |
|  | Without ACEI/ARB | 11.93 | 3.16-45.00 | <0.001 |
|  | With vasopressor | 12.76 | 3.48-46.72 | <0.001 |
|  | Constant | 0.00 |  | <0.001 |
| **7** | Age >79 years | 10.91 | 2.66-44.73 | 0.001 |
|  | NT-pro BNP>8100 pg/ml | 5.08 | 1.43-18.05 | 0.012 |
|  | With MV support | 7.81 | 1.96-31.10 | 0.004 |
|  | With NIPPV support | 6.49 | 1.59-26.51 | 0.009 |
|  | Without ACEI/ARB | 14.04 | 3.43-57.54 | <0.001 |
|  | Experience of CPR | 11.88 | 1.64-86.04 | 0.014 |
|  | With vasopressor | 9.49 | 2.35-38.33 | 0.002 |
|  | Constant | 0.00 |  | <0.001 |
| **8** | Age >79 years | 11.78 | 2.57-54.05 | 0.002 |
|  | NT-pro BNP>8100 pg/ml | 5.49 | 1.47-20.51 | 0.011 |
|  | With MV support | 10.99 | 2.49-48.45 | 0.002 |
|  | With NIPPV support | 6.99 | 1.60-30.67 | 0.010 |
|  | Without ACEI/ARB | 12.61 | 2.87-55.39 | 0.001 |
|  | Without beta-blocker | 12.53 | 1.29-121.92 | 0.029 |
|  | Experience of CPR | 11.15 | 1.43-86.90 | 0.021 |
|  | With vasopressor | 9.16 | 2.06-40.67 | 0.004 |
|  | Constant | 0.00 |  | <0.001 |
| **9** | Age >79 years | 12.69 | 2.60-61.86 | 0.002 |
|  | NT-pro BNP>8100 pg/ml | 6.65 | 1.71-25.95 | 0.006 |
|  | With MV support | 9.47 | 2.06-43.54 | 0.004 |
|  | With NIPPV support | 6.18 | 1.35-28.21 | 0.019 |
|  | Without ACEI/ARB | 9.49 | 2.13-42.21 | 0.003 |
|  | Without beta-blocker | 17.75 | 1.59-198.65 | 0.020 |
|  | Without loop diuretics | 4.01 | 1.14-14.08 | 0.030 |
|  | Experience of CPR | 14.39 | 1.63-127.28 | 0.017 |
|  | With vasopressor | 8.01 | 1.77-36.17 | 0.007 |
|  | Constant | 0.00 |  | <0.001 |

**Note:** The variables put into the logistic regression model were the 17 predictors which exhibited significant correlation with in-hospital mortality in Pearson’s correlation test (shown in Table 2). Finally, 9 independent predictors of in-hospital mortality were exhibited.

**Abbreviations:** ACEI=Angiotensin converting enzyme inhibitors, ARB=Angiotensin receptor blocker, CI=Confidence interval, CPR=Cardiopulmonary Resuscitation, Na=Serum sodium, NIPPV=Noninvasive Positive Pressure Ventilators; OR= odd’s ratio**.**

**Supplementary table 2. Steps of determining independent predictors for in-hospital mortality, model 2.**

| **Step** | **Predictors** | **Adjusted OR** | **95% CI** | **p-value** |
| --- | --- | --- | --- | --- |
| **1** | HR > 140 bpm | 6.25 | 1.60-24.44 | 0.008 |
|  | CCR < 60 ml/min/1.73m2 | 2.90 | 1.02-8.19 | 0.045 |
|  | Na < 130 mEq/L | 3.74 | 1.47-9.47 | 0.005 |
|  | Age >79 years | 2.50 | 1.09-5.73 | 0.030 |
| **2** | HR > 140 bpm | 5.25 | 1.33-20.74 | 0.018 |
|  | CCR < 60 ml/min/1.73m2 | 2.16 | 0.73-6.35 | 0.163 |
|  | Na < 130 mEq/L | 3.60 | 1.38-9.36 | 0.009 |
|  | Age >79 years | 2.40 | 1.03-5.59 | 0.042 |
|  | NT-pro BNP>8100 pg/ml | 3.23 | 1.26-8.27 | 0.015 |
| **3** | HR > 140 bpm | 6.82 | 1.30-35.93 | 0.023 |
|  | CCR < 60 ml/min/1.73m2 | 1.64 | 0.50-5.38 | 0.417 |
|  | Na < 130 mEq/L | 2.51 | 0.79-8.03 | 0.120 |
|  | Age >79 years | 2.77 | 1.03-7.47 | 0.045 |
|  | NT-pro BNP>8100 pg/ml | 3.86 | 1.31-11.35 | 0.014 |
|  | Without ACEI/ARB | 0.21 | 0.06-0.72 | 0.014 |
|  | Without beta-blocker | 0.07 | 0.01-0.62 | 0.017 |
|  | Without loop diuretics | 0.17 | 0.06-0.48 | 0.001 |
| **4** | HR > 140 bpm | 4.81 | 0.57-40.68 | 0.150 |
|  | CCR < 60 ml/min/1.73m2 | 1.73 | 0.37-8.02 | 0.484 |
|  | Na < 130 mEq/L | 2.19 | 0.54-8.95 | 0.276 |
|  | Age >79 years | 12.69 | 1.33-20.20 | 0.001 |
|  | NT-pro BNP>8100 pg/ml | 6.65 | 0.67-4.81 | 0.002 |
|  | Without ACEI/ARB | 9.49 | 0.96-20.67 | 0.002 |
|  | Without beta-blocker | 17.75 | 1.06-33.38 | 0.003 |
|  | Without loop diuretics | 4.01 | 0.11-4.04 | 0.025 |
|  | With NIPPV support | 6.18 | -0.41-4.84 | 0.028 |
|  | With MV support | 9.47 | 0.79-5.11 | 0.001 |
|  | With vasopressor | 8.01 | 0.863-5.46 | <0.010 |
|  | Experience of CPR | 14.39 | 0.53-22.02 | 0.003 |

**Note:** The variables put into the logistic regression model were the 3 predictors (HR> 140 bpm, CCR< 60 ml/min/1.73m2, and Na < 130 mEq/L) which were previously exhibited as predictors for mortality in addition to the 9 independent predictors (shown in table 3) disclosed in the current study. It was apparent that the predictive power of NT-pro BNP>8100 pg/ml was larger than the previously mentioned predictors.

**Abbreviations:** ACEI=Angiotensin converting enzyme inhibitors, ARB=Angiotensin receptor blocker, CI=Confidence interval, HR=Heart Rate, CCR= Creatinine Clearance Rate, CPR=Cardiopulmonary Resuscitation, MV=mechanical ventilator Na=Serum sodium, NIPPV=Noninvasive Positive Pressure Ventilators; OR= odd’s ratio

**Supplementary figure 1. Seeking best cut-off point by** **generalized additive models**

Note: The plot of generalized additive models (GAM) for using their best cut points for measuring the probability of in-hospital mortality revealed that the probability of death initiated to elevate since NT-pro-BNP level of 0 pg/ml, and the best cut-off point of NT-pro-BNP level was 8100 pg/ml.

**
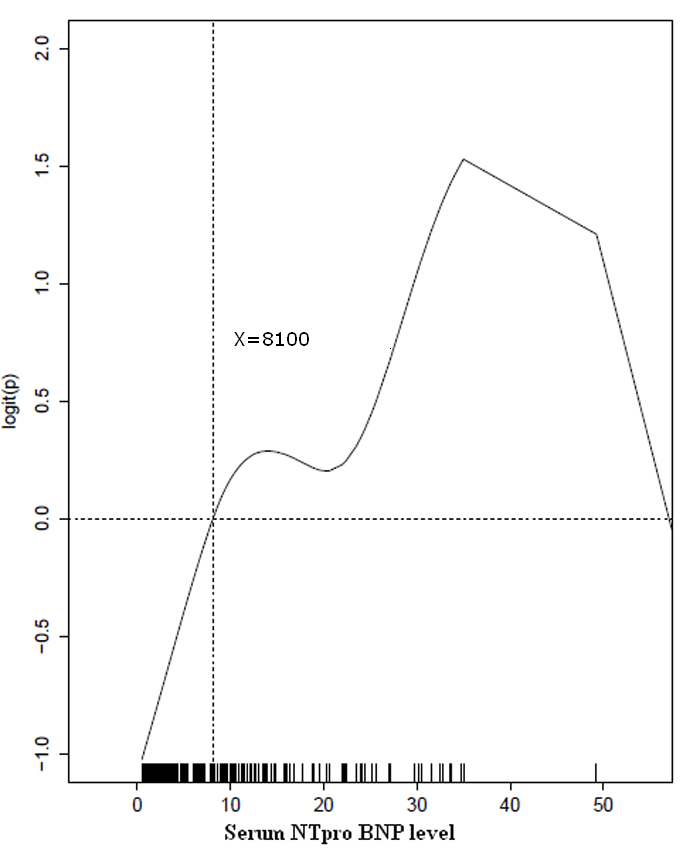
**

**Supplementary figure 2. The plot of change in deviance for testing the logistic regression predicting in-hospital mortality**

Note: The plot of the change in deviance was used to identify cases that are poorly fit by the model and demonstrated that only a few cases dispersed from the lines, indicating the good fit of the proposed model.


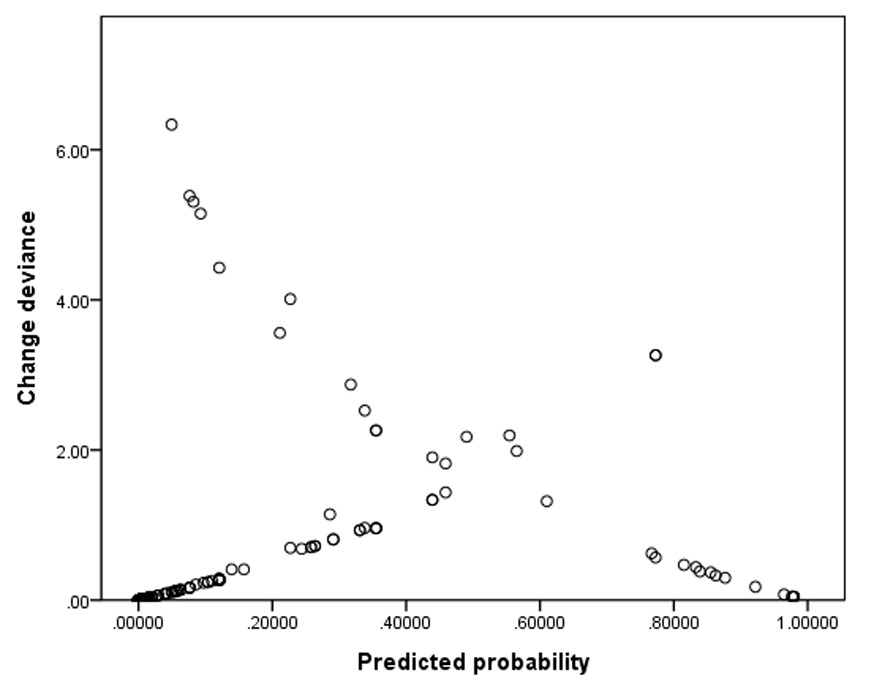


**Supplementary figure 3. Predictability for in-hospital mortality of NT-pro BNP-based score by heart failure with a reduced ejection fraction (HFrEF) and heart failure with a preserved ejection fraction (HFpEF) groups**

Note: **Solid line** denotes **HFrEF** group, with an Area Under the Curve (AUC) of 0.96 (95% Confidence Interval (CI): 0.94 - 1.00, *p* <0.0001); **Dashed line** denotes **HFpEF** group, with an AUC of 0.96 (95% CI: 0.91 - 0.99, *p*< 0.001).


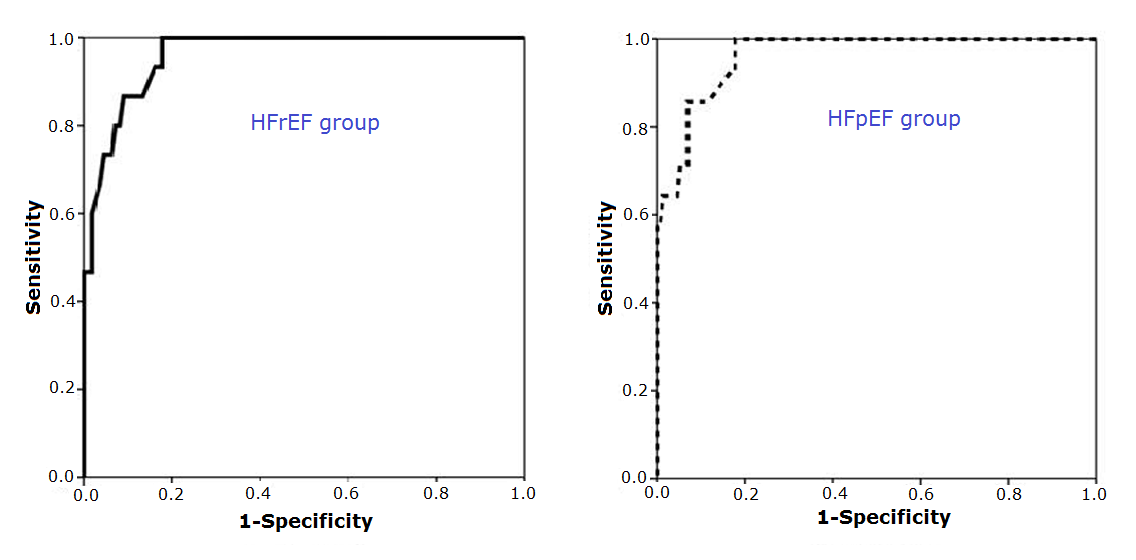

Supplement: Supplementary Information [file srep29590-s1.doc]
